# Uncoupling Protein 2 and Peroxisome Proliferator-Activated Receptor *γ* Gene Polymorphisms in Association with Diabetes Susceptibility in Chinese Han Population with Variant Glucose Tolerance

**DOI:** 10.1155/2018/4636783

**Published:** 2018-04-05

**Authors:** Meicen Zhou, Shuli He, Fan Ping, Wei Li, Lixin Zhu, Xiangli Cui, Linbo Feng, Xuefeng Zhao, Huabing Zhang, Yuxiu Li, Qi Sun

**Affiliations:** ^1^Department of Endocrinology, Key Laboratory of Endocrinology, Ministry of Health, Peking Union Medical College Hospital, Beijing 100730, China; ^2^Department of Endocrinology, Beijing Jishuitan Hospital, Beijing 100035, China; ^3^Department of Nutrition, Peking Union Medical College Hospital, Beijing 100730, China; ^4^Nankou Community Health Service Centers, Changping District, Beijing 102200, China; ^5^Nankou Railway Hospital, Changping District, Beijing 102200, China

## Abstract

**Objective:**

To investigate the association of polymorphisms in uncoupling protein 2 (UCP2) and peroxisome proliferator-activated receptor (PPAR*γ*) with glucolipid metabolism in Chinese Han population.

**Methods:**

Five hundred eighty-nine subjects were divided into normal glucose tolerance (NGT) group (*n* = 198) and abnormal glucose tolerance group (*n* = 358). HbA1c, blood lipid profile, plasma glucose, and insulin were determined. Insulin sensitivity (HOMA-IR and Matsuda index (ISI_M_)) and insulin secretion indexes (HOMA-*β*, early and total phase disposition index) were evaluated. Eight potential functional SNPs in UCP2 and 7 in PPAR*γ* were selected. SNPs were genotyped on Sequenom MassARRAY platform.

**Results:**

The GG genotype of rs2920502 in PPAR*γ* was associated with decreased risk of impaired glucose tolerance (G allele: OR: 0.818, 95%CI: 0.526–0.969, *P* = 0.042; GG: OR: 0.715, 95%CI: 0.527–0.97, *P* = 0.031). The TT genotype of rs3856806 in PPAR*γ* was associated with increased risk of impaired glucose tolerance (T allele: OR: 1.46, 95%CI: 1.055–2.017, *P* = 0.022; TT: OR: 1.58, 95%CI: 1.104–2.761, *P* = 0.032). The GG genotype of rs2920502 in PPAR*γ* had better blood glucose and increased insulin secretion and had lower HOMA-IR than GC/CC genotypes.

**Conclusion:**

It probably could prevent insulin resistance in early stage by classifying the genotype of rs649446 and rs7109266 in UCP2. The GG genotype of rs2920502 in PPAR*γ* had a decreased risk for diabetes. The TT genotype of rs3856806 in PPAR*γ* had an increased risk for diabetes.

## 1. Introduction

Uncoupling protein 2 (UCP2), which is widely expressed in human tissues and serves as an uncoupler of oxidative phosphorylation, is involved in the regulation of glucolipid metabolism and ATP production [1, 2]. The association of the polymorphisms in UCP2 with diabetes and obesity have been widely evaluated, most studies focused on Ala55Val (rs660339) in exon 4, 45 bp insertion/deletion in exon 8, and -866G/A (rs659336) in the promoter region [3, 4]. The polymorphisms in UCP2 regulate the expression of mRNA and protein, which have vital effects on islet *β*-cell function and insulin sensitivity [5, 6]. The -866AA genotype carriers have decreased glucose-stimulated insulin secretion and have increased risk of diabetes than those GG genotype carriers [7]. Although a variant allele of the Ala55Val polymorphism was reported to be associated with lower energy expenditure and the 45 bp insertion/deletion polymorphisms were found to be functional on mRNA expression, the association of Ala55Val (rs660339) in exon 4 with diabetes remain controversial [8–10].

Peroxisome proliferator-activated receptor (PPARs) play pivotal roles in the control of the transcription of UCP2 [11, 12]. PPARs have three isoforms, including Ppar*α*, Ppar*δ*, and PPAR*γ*. PPAR*γ* is a regulator of lipid and glucose metabolism and therefore its synthetic ligands such as glitazone—the derivative of thiazolidinediones (e.g., troglitazone, rosiglitazone, and pioglitazone)—improve insulin and glucose parameters and increase whole body insulin sensitivity [13]. These PPAR*γ* synthetic ligands could indirectly increase insulin-stimulated glucose uptake in adipocytes, skeletal muscle cells, and hepatocytes [13]. Our previous study found that the UCP2-deficient mice fed with a long-term high-fat diet had better insulin sensitivity, improved lipid metabolism, and upregulated expression of PPAR*γ* in PPAR signaling pathway, which suggested the ameliorated lipid metabolism and insulin sensitivity in UCP2-deficient mice probably via PPAR*γ*. It was most likely that among Ppar isoforms, PPAR*γ* was the major regulator of UCP2 in high-fat diet [14]. One study based on Chinese Han population showed that functional SNPs of PPAR*γ* were associated with MetS [15]. The relationship between potential functional SNPs and diabetes remains unknown.

The inflammation pathway is involved in the pathophysiology of diabetes and obesity. Previous study showed that PPAR polymorphisms were independently associated with CRP levels in Chinese Han population; PPARs polymorphisms interact with overweight/obesity to set CRP levels [16]. In healthy children and adolescents, UCP2 -866G>A modified low-grade inflammatory state [17]. Whether UCP2 and PPAR*γ* polymorphisms have an effect on inflammation state in diabetes remains unknown.

In this study, we built a Chinese Han population cohort with variant glucose tolerance and aimed to further investigate the association of polymorphisms in the functional region of UCP2 and PPAR*γ* with glucolipid metabolism.

## 2. Subjects and Methods

### 2.1. Subjects

All subjects were recruited from a type 2 diabetes project in a Beijing suburb in China between March 2014 and January 2015. Five hundred eighty-nine subjects without a history of diabetes underwent a 75 g OGTT. The 75 g OGTT was conducted after an overnight fast (>10 hours). Blood samples were collected at 0 minutes, 30 minutes, 60 minutes, and 120 minutes following the OGTT. The glucose tolerance status of each subject was classified based on the 1999 criteria of the WHO: a normal glucose tolerance (NGT), indicated by fasting plasma glucose (FPG) < 6.1 mmol/l and 2 h postprandial glucose (2 h PG) < 7.8 mmol/l; prediabetes, indicated by impaired fasting glucose (IFT): 6.1 mmol/l ≤ FPG < 7.0 mmol/l and 2 h PG < 7.8 mmol/l; impaired glucose tolerance (IGT), indicated by FPG < 6.1 mmol/l and 7.8 ≤ 2 h PG < 11.1 mmol/l; or IFT + IGT, with T2DM indicated by FPG ≥ 7.0 mmol/l or 2 h PG ≥ 11.1 mmol/l.

The subjects who have a current history of cigarette smoking and alcohol drinking were excluded, and subjects with serious diseases such as heart disease, stroke, kidney disease, liver disease, and inflammatory disease were also excluded. Subjects who were on steroids or who were taking drugs interfering with lipid metabolism such as lipid-lowering agents, diuretics, *β*-blockers, and fish oil were excluded. On the basis of the 75 g OGTT results, subjects were divided into normal glucose tolerance (NGT) group (*n* = 198) and abnormal glucose tolerance group (*n* = 358). The study protocol was approved by the Ethics Committee of Peking Union Medical College Hospital. The subjects voluntarily signed informed consent forms.

### 2.2. Clinical Measurement

A standardized medical history and accurate physical examination were undertaken in all of the subjects before a 75 g OGTT was administered. Measurements of waist circumference (WC) (midway between the iliac crest and the costal margin) and hip circumference (HC) (at the level of the trochanters) were performed twice by the same observer, and the mean value was recorded. Weight and height were measured without shoes in light clothing, and body mass index (BMI) was calculated by dividing the body weight in kilograms by the square of the height in meters. Blood pressure measurements were obtained twice with a standard mercury sphygmomanometer with the subjects at rest, and the mean value was calculated.

### 2.3. Biochemical Measurements

Plasma glucose was measured by glucose oxidase assay. TC, TG, HDL-C, and LDL-C were determined using an automated analyzer. Serum insulin and C peptide were measured by chemiluminescent enzyme immunoassay. HbA1c analysis was performed by high-performance liquid chromatography (intra-assay CV < 3%, interassay CV < 10%).

### 2.4. Assessment of IR

Homeostatic model assessment of insulin resistance (HOMA-IR) was calculated to evaluate the IR [18].

### 2.5. Assessment of *β*-Cell Function

The homeostasis model assessment of insulin secretion (HOMA-*β*) was calculated as basal insulin release [18]. Early-phase insulin release was calculated as the total insulin area under the curve divided by the total glucose area under the curve during the first 30 min of the OGTT (InsAUC_30_/GluAUC_30_), which was shown to have a strong correlation with first-phase insulin secretion [19]. Insulin secretion relative to insulin sensitivity (ISI_M_: Matsuda insulin sensitivity index) was expressed as the disposition index (DI), calculated as early-phase DI_30_ = [InsAUC_30_/GluACU_30_] × ISI_M_, (ΔIns30/ΔGlu30)/HOMA-IR and total-phase DI_120_ = [InsAUC_120_/GluACU_120_] × ISI_M_. Another formula for assessing early-phase insulin release was (ΔIns_30_/ΔGlu_30_)/HOMA-IR.

### 2.6. Measurement of Tumor Necrosis Factor-*α* (TNF-*α*) and Interleukine-6 (IL-6)

Serums were from fasting blood samples. The levels of TNF-*α* and IL-6 were performed as per the manufacturer's instructions (Cloud-Clone Corp., Houston, USA), and absorbance kinetics was measured through an ELISA reader.

### 2.7. SNP Selection, Genotyping, and Genotype Quality Control

Genomic DNA was extracted from peripheral blood samples using the QIAamp DNA blood mid kit (Qiagen, Hilden, Germany); purified DNA samples were diluted and quantified using a NanoDrop 1000 spectrophotometer (Thermo Fisher Scientific, Wilmington, DE, USA). We selected 8 potential functional SNPs of UCP2 and 7 potential functional SNPs of PPAR*γ*, including promoter, exon, 5′ untranslated region and 3′ untranslated region based on the screening standards (the minor allele frequencies (MAF) are more than 20% in Han Chinese according to the HapMap Han Chinese in Beijing (CHB) group). Further, we reviewed the documents about the selected SNPs and forecasted their function according to NIH SNPinfo Web Server (http://snpinfo.niehs.nih.gov/) (Table 1). The MAFs of the selected SNPs in the study were listed in Table 2. All candidate SNPs were genotyped on Sequenom MassARRAY platform.

### 2.8. Statistical Analysis

Continuous variables were expressed as mean ± standard deviations (SD). Statistical significances for continuous variables were assessed using Student's *t*-test and for categorical variables using chi-square test. Hardy-Weinberg equilibrium tests were performed using Pearson's chi-square for each SNP among control subjects. One-way ANOVA was used to compare different genotypes of every SNP site for continuous variables. All the statistical analyses were performed using SPSS 19.0 for windows and SAS 9.2 (SAS Institute) and a *P* value of <0.05 was considered statistically significant.

## 3. Results

### 3.1. Allele Frequency Analysis

All loci conformed to Hardy-Weinberg equilibrium as shown in Supplementary Table 1. There was no significant difference in allele frequency of each SNP in UCP2 between prediabetes/diabetes group and normal glucose tolerance group (Table 3). In PPAR*γ*, the G allele in rs2920502 decreased the risk of diabetes (OR: 0.818, 95%CI: 0.526–0.969, *P* = 0.042), the T allele in rs3856806 increased the risk of diabetes (OR: 1.46, 95%CI: 1.055–2.017, *P* = 0.022) (Table 3). In UCP2, there was no significant difference between alleles in each SNP.

### 3.2. Genotype Analysis

The association of SNPs with prediabetes/diabetes was assessed by crosstab test and logistic regression after adjustment for age and sex. In PPAR*γ*, the frequency of GG genotype in rs2920502 was significantly lower in prediabetes/diabetes subjects (6.85%) than in the normal glucose tolerance subjects (12.69%); logistic regression analysis revealed that subjects with GG genotype of rs2920502 in PPAR*γ* had less risk for prediabetes/diabetes compared to CC genotype (odd ratio (OR): 0.715; 95% confidence interval (CI): 0.527–0.97, *P* = 0.031). The frequency of TT genotype in rs3856806 was significantly higher in prediabetes/diabetes subjects than in the normal glucose tolerance subjects; logistic regression analysis showed that subjects with TT genotype of rs3856806 in PPAR*γ* had higher risk for diabetes compared to CC (OR: 1.58, 95%CI: 1.104–2.761, *P* = 0.032). Furthermore, we, respectively, performed a logistic regression analysis under a recessive inheritance model (GG versus GC + CC) in rs2920502 and a dominant inheritance model (TT + TC/CC) in rs3856806; the regression showed that the odd ratio for GG versus GC + CC in rs2920502 was 0.506 (95%CI: 0.282–0.906, *P* = 0.022) and the odd ratio for TT + TC/CC in rs3856806 was 1.479 (95%CI: 1.026–2.133, *P* = 0.036). These were in accordance with the allele frequency analysis, which implied that G allele carriers in rs2920502 were less susceptible to develop diabetes and T allele carriers in rs3856806 were more susceptible to develop diabetes. No significant difference was found at other loci in PPAR*γ* (Table 4). There was no significant difference in the genotype of each SNPs in UCP2 (Table 4).

### 3.3. Haplotype Analysis

There was a linkage disequilibrium in PPAR*γ* and UCP2, respectively. The haplotype frequency distribution of each gene between prediabetes/diabetes and normal glucose tolerance was summarized in Table 5; however, haplotype frequency was not significantly different between prediabetes/diabetes and control.

### 3.4. Association of Genotype with Demographic Characteristics

In UCP2, the waist-to-hip ratio in subjects with AA genotype of rs7109266 were higher than that in subjects with GG or GA genotype, but age, BMI, and blood pressure were not different among genotypes of other SNPs (Table 6). Age, BMI, blood pressure, and waist-to-hip ratio were not different among genotypes of selected SNPs in PPAR*γ* (Table 7).

### 3.5. Association of Genotype with Insulin Secretion Function, Blood Glucose, and Lipid Profiles

Subjects with TT genotype of rs649446 or with AA genotype of rs7109266 in UCP2 had higher fasting insulin, HOMA-IR, and HOMA-*β* than subjects with other genotypes, but blood glucose profiles including fasting and 2 hr postprandial glucose were not significantly different among genotypes (Table 8). There was no significant difference in glucose profiles and insulin secretion in other loci of UCP2 (Table 8). The serum lipid TC, TG, HDL-C, and LDL-C were not significantly different among genotypes of selected SNPs in UCP2 (Table 9).

Subjects with GG genotype of rs2920502 in PPAR*γ* had better HbA1c, 0 min, 30 min, and 120 min blood glucose, increased 60 min and 120 min insulin secretion after taking 75 g glucose, and lower serum TC, TG, and LDL-C compared to GC/CC genotypes (Table 10); the HOMA-IR in GG genotype was lower than GC/CC genotypes. Subjects with TT genotype of rs2920503 in PPAR*γ* had better HbA1c, 0 min, 30 min, 60 min, and 120 min blood glucose and had increased serum insulin in 120 min after taking 75 g glucose compared to TC/CC genotypes (Table 10). Subjects with TT genotypes of rs3856806 had higher fasting blood glucose than TC/CC genotypes, and postprandial blood glucose and insulin secretion were not significantly different among genotypes. The blood glucose at 0 min, 30 min, 60 min, and 120 min after taking 75 g glucose in subjects with AA/GG genotype of rs79310821 were better than subjects with GA genotype. The blood glucose at 0 min, 30 min, 60 min, and 120 min after taking 75 g glucose in subjects with TT/GG genotype of rs79310821 was better than that in subjects with TG genotype, and index of insulin secretion-HOMA-*β*, DI30, and DI120 were higher in TT/GG genotype than in TG genotype. The serum lipid profiles were not significantly different in other loci in PPAR*γ* (Table 11).

### 3.6. Association of Genotype with Inflammation

There was no significant difference in TNF-*α* among genotypes in UCP2. The serum IL-6 was higher in subjects with TT genotype of rs660339 than in GG/GA genotype, and IL-6 was higher in subjects with TT genotype of rs649446 than in CC/TC genotype (Table 12). There was no significant difference in inflammation indicators among genotypes in PPAR*γ* (Table 13).

## 4. Discussion

The effects of UCP2 on proton leakage and the decline in ATP synthesis in *β*-cells show that this protein is a negative regulator of insulin secretion. Increased expression of UCP2 results in decreased ATP synthesis, which inhibits ATP-sensitive potassium (K-ATP) channels, leading to the decline of glucose-stimulated insulin secretion [1]. Our previous study showed that UCP2 deficiency led to the amelioration of lipid metabolism and improved blood glucose by simultaneously promoting insulin sensitivity and *β*-cell function [1, 2]. Obesity and T2DM closely associated with SNPs in UCP2, including rs660339 (Ala55Val), rs659366 (-866G/A), and rs591758 [7]. In this study based on Chinese Han population in Beijing district, we selected 8 SNPs in the functional region of UCP2, and the results indicated that the alleles and genotypes were not significantly different between prediabetes/diabetes and control. Further genotype and clinical features analysis showed that subjects with TT genotype of rs649446 or subjects with AA genotype of rs7109266 in UCP2 had higher HOMA-IR and HOMA-*β*, subjects with AA genotype of rs7109266 also had higher waist-to-hip ratio, which suggested that subjects with TT genotype of rs649446 or subjects with AA genotype of rs7109266 were more susceptible to develop insulin resistance. Previous study showed that human islets with GA genotype of UCP2-866 polymorphism have decreased glucose-stimulated insulin secretion compared to GG genotype islets [3]. However, the pathway between UCP2 polymorphism and HOMA index has not been elaborated clearly. The study was the first one to investigate the association of the above SNPs with insulin resistance in Chinese Han population in Beijing district, it probably could give certain suggestion to prevent insulin resistance in early stage by classifying the genotype of the above SNPs inUCP2.

The inflammation pathway is involved in the pathophysiology of diabetes and obesity. The study indicated that subjects with GG/GA genotype of rs660339 in UCP2 had higher serum IL-6 levels than those with AA genotype, and subjects with TT genotype of rs649446 had higher IL-6 than those with CC/TC genotypes. IL-6 is a central player in the regulation of inflammation, leading to insulin resistance. Its quantitative release from adipose tissue results in a subclinical and systemic elevation of IL-6 plasma levels with increasing body fat content, which may be implicated in the proinflammatory state leading to insulin resistance [20]. On the other hand, IL-6 produced in the working muscle during physical activity could act as an energy sensor by activating AMP-activated kinase and enhancing glucose disposal, lipolysis, and fat oxidation. In addition, both impaired IL-6 secretion and action are risk factors for weight gain [21]. Previous study suggested that people with GG/GA genotype of rs660339 in UCP2 had an increased risk for diabetes, obesity, and metabolic syndrome; the elevated IL-6 in the subjects with GG/GA genotype suggested that these kinds of SNP was closely related to inflammation, which play an important role in the mechanism of diabetes and its complications.

PPAR*γ*, which is a central nuclear receptor, is involved in fatty acid and glucose metabolism and is closely associated with insulin sensitivity. In clinical work, PPAR*γ* agonist glitazone—the derivative of thiazolidinediones—could improve insulin resistance by indirectly increasing insulin-stimulated glucose uptake in adipocytes, skeletal muscle cells, and hepatocytes and inhibiting proinflammation cytokines produced from mononuclear macrophages [22]. Our previous study showed that UCP2 deficiency could improve insulin sensitivity and *β*-cell function by PPAR signaling pathway. PPAR*γ* regulates UCP2 in the condition of a high-fat diet [14]. Among the selected 7 SNPs of PPAR*γ* in our study, two loci (rs2920502 and rs3856806) were reported to be related to glucolipid metabolism [22]. This study suggested that subjects with GG genotype of rs2920502 in PPAR*γ*, who had better early- and total-stage insulin secretion function and better serum lipid condition, had a decreased risk for diabetes in Chinese Han population of Beijing district. Prakash et al. reported that in Nanjing and Southwest district of China, GG genotype of rs2920502 was a protective factor for metabolism syndrome, GG carriers had elevated serum adiponectin, which is a kind of anti-inflammatory and antiatherosclerosis cytokine that could prevent metabolism syndrome; therefore, GG genotype of rs2920502 probably improved glucolipid metabolism by regulating the secretion of adiponectin [22]. In our study, subjects with TT genotype of rs3856806 in PPAR*γ* had an increased risk for diabetes, and the result was in accordance with a previous study based on Chinese Han population; however, studies based on Indians and Singaporeans showed that TT genotype of rs3856806 could decrease the risk for diabetes. Evidence also showed that rs3856806 in PPAR*γ* had a close relationship with metabolic syndrome, subjects with TT genotype had higher BMI in males, and those with TT/TC genotypes had higher systolic blood pressure, HOMA-IR, and larger body fat percentage, which were all related to insulin sensitivity. For that reason, rs3856806 was considered as the vital regulation loci of insulin sensitivity.

In our study based on Chinese Han population in Beijing district, the sample size was limited; we found that the alleles and genotypes of rs2920503, rs73813168, rs79310821, rs73021485, and rs1702907 in PPAR*γ* had no significant difference between prediabetes/diabetes and normal glucose tolerance, but the genotype-phenotype analysis suggested that subjects with TT genotype of rs2920503 had better insulin secretion function and blood glucose status and subjects with AA/GG genotypes of rs79310821 or with TT/GG genotypes of rs73021485 had better blood glucose status. Studies with a larger sample size are needed to confirm the association of SNPs in PPAR*γ* with diabetes.

In summary, this study investigated the association of polymorphism of UCP2 and PPAR*γ* with glucolipid metabolism based on Chinese Han population in Beijing district; it probably could give certain suggestions to prevent insulin resistance in the early stage by classifying the genotype of rs649446 and rs7109266 in UCP2. The polymorphism of PPAR*γ* closely associated with glucolipid metabolism. Subjects with GG genotype of rs2920502 in PPAR*γ*, who had better early- and total-stage insulin secretion function and better serum lipid condition, had a decreased risk for diabetes. Subjects with TT genotype of rs3856806 in PPAR*γ* had an increased risk for diabetes.

## Figures and Tables

**Table 1:** The selected functional SNPs of UCP2 and PPAR*γ*.

Gene name	SNP number	Function	Minor allele frequency	Data sources	Relevant documents	Function forecast
UCP2	rs660339	Missense	0.422	HapMap	Investigation of variants in UCP2 in Chinese type 2 diabetes and diabetic retinopathy	Splicing (ESE or ESS)/nsSNP
	rs659366	Promoter	0.442	HapMap	The common -866G/A polymorphism in the promoter region of the UCP-2 gene is associated with reduced risk of type 2 diabetes in Caucasians from Italy	TFBS
	rs649446	Promoter	0.35	HapMap	No report	TFBS
	rs586773	5′ near	0.476	1000 Genomes	No report	TFBS
	rs34408426	5′ near	0.476	1000 Genomes	No report	TFBS
	rs7109266	5′ near	0.349	HapMap	No report	TFBS
	rs3019463	5′ near	0.476	1000 Genomes	No report	TFBS
	rs591758	5′ near	0.422	HapMap	Genetic variants in the UCP2-UCP3 gene cluster and risk of diabetes in the Women's Health Initiative Observational Study	TFBS

PPAR*γ*	rs3856806	Cds-synon	0.233	HapMap	Gene-gene interactions among PPAR*α*/*δ*/*γ* polymorphisms for hypertriglyceridemia in Chinese Han population	—
	rs2920502	Promoter	0.244	HapMap	Genetic variants in peroxisome proliferator-activated receptor-*γ* and retinoid X receptor-*α* gene and type 2 diabetes risk: a case-control study of a Chinese Han population	TFBS
	rs17029007	5′ UTR	0.102	1000 Genomes	No report	TFBS/splicing
	rs73021485	Promoter	0.374	1000 Genomes	No report	—
	rs73813168	5′ near	0.103	1000 Genomes	No report	—
	rs2920503	5′ near	0.32	1000 Genomes	No report	—
	rs79310821	5′ near	0.371	1000 Genomes	No report	

TFBS: transcription factor binding site.

**Table 2:** The MAFs of the selected SNPs in the study.

Chromosome	Gene name	SNP number	Alleles	Detection rate (%)	MAF in CHB	MAF in the study
11	UCP2	rs660339	A/G	99.8	0.42	0.47
		rs659366	T/C	99.8	0.44	0.48
		rs649446	T/C	99.8	0.35	0.34
		rs586773	T/A	98.9	0.48	0.48
		rs34408426	G/A	99.3	0.48	0.48
		rs7109266	A/G	99.8	0.35	0.33
		rs3019463	T/C	97.4	0.48	0.48
		rs591758	C/G	99.8	0.42	0.48

3	PPAR*γ*	rs2920503	A/G	97.7	0.32	0.31
		rs73813168	G/A	98.5	0.10	0.12
		rs79310821	A/G	99.2	0.37	0.35
		rs73021485	T/G	99.7	0.37	0.35
		rs2920502	G/C	99.3	0.24	0.31
		rs17029007	A/G	98.9	0.10	0.13
		rs3856806	T/C	99.5	0.23	0.20

MAF: minor allele frequency; CHB: Han Chinese in Beijing.

**Table 3:** Allele frequency analysis between prediabetes/diabetes group and normal blood glucose group.

Gene name	SNP number	Allele	Prediabetes/diabetes group	Normal blood glucose group	Odd ratio (OR)	95% confidence interval (CI)		*P* value
			(*N*)	(*N*)		Low	High	
UCP2	rs660339	A	346	187	0.997	0.775	1.281	0.9778
		G	388	209				
	rs659366	T	351	192	0.973	0.758	1.248	0.828
		C	383	204				
	rs649446	T	244	136	0.949	0.728	1.238	0.700
		C	490	260				
	rs586773	T	351	192	0.959	0.750	1.227	0.741
		A	381	200				
	rs34408426	G	350	193	0.959	0.750	1.227	0.738
		A	380	201				
	rs7109266	A	240	135	0.935	0.715	1.222	0.624
		G	494	261				
	rs3019463	T	343	188	0.978	0.763	1.253	0.861
		C	373	200				
	rs591758	C	352	194	0.959	0.750	1.227	0.738
		G	382	202				

PPAR*γ*	rs2920503	A	213	126	0.918	0.708	1.191	0.519
		G	499	270				
	rs73813168	G	80	58	0.693	0.474	1.012	0.057
		A	646	334				
	rs79310821	A	265	126	1.2	0.924	1.560	0.172
		G	467	266				
	rs73021485	T	271	127	1.241	0.957	1.609	0.104
		G	463	269				
	rs2920502	G	214	132	0.818	0.526	0.969	0.042^∗^
		C	516	262				
	rs17029007	A	81	60	0.699	0.486	1.005	0.053
		G	645	336				
	rs3856806	T	163	66	1.460	1.055	2.017	0.022^∗^
		C	567	330				

^∗^ *P* < 0.05.

**Table 4:** Genotype analysis between prediabetes/diabetes group and normal blood glucose group.

Gene name	SNP number	Genotype	Prediabetes/diabetes group	Normal blood glucose group	Odd ratio (OR)	95% confidence interval (CI)		*P* value
						Low	High	
UCP2	rs660339	AA	77	42	0.996	0.774	1.28	0.9781
		AG	192	103				
		GG	98	53				
	rs659366	TT	80	45	0.972	0.757	1.247	0.968
		TC	191	102				
		CC	96	51				
	rs649446	TT	38	19	1.003	0.739	1.362	0.699
		TC	168	98				
		CC	161	81				
	rs586773	TT	83	47	0.959	0.749	1.226	0.9377
		TA	185	98				
		AA	98	51				
	rs34408426	GG	83	47	0.959	0.749	1.226	0.9444
		GA	184	99				
		AA	98	51				
	rs7109266	AA	36	18	0.997	0.729	1.363	0.631
		AG	168	99				
		GG	163	81				
	rs3019463	TT	82	45	0.979	0.763	1.255	0.9793
		TC	179	98				
		CC	97	51				
	rs591758	CC	83	47	0.959	0.749	1.226	0.9443
		CG	186	100				
		GG	98	51				

PPAR*γ*	rs2920503	AA	34	23	0.891	0.665	1.193	0.438
		AG	145	80				
		GG	177	95				
	rs73813168	GG	2	2	0.701	0.262	1.877	0.480
		AA	285	140				
		AG	76	54				
	rs79310821	AA	44	23	1.108	0.834	1.471	0.480
		AG	177	80				
		GG	145	93				
	rs73021485	TT	47	23	1.159	0.875	1.535	0.304
		GG	143	94				
		GT	177	81				
	rs2920502	GG	25	25	0.715	0.527	0.97	0.031^∗^
		CC	176	90				
		CG	164	82				
	rs17029007	AA	5	3	0.905	0.439	1.864	0.786
		AG	71	54				
		GG	287	141				
	rs3856806	TT	16	3	1.58	1.104	2.761	0.032^∗^
		CC	218	136				
		CT	131	58				

^∗^ *P* < 0.05.

**Table 5:** The haplotype frequency distribution between prediabetes/diabetes and normal glucose tolerance.

Gene name	Haplotype	Prediabetes/diabetes, normal glucose tolerance frequency	*χ* ^2^	*P* value
UCP2	GCCAAGCG	0.511, 0.513	0.003	0.953
	ATTTGATC	0.318, 0.329	0.133	0.715
	ATCTGGTC	0.146, 0.143	0.021	0.884

PPAR*γ*	CAATCG	0.362, 0.316	2.473	0.1158
	TAGGCG	0.307, 0.318	0.149	0.6992
	CAGGGG	0.182, 0.184	0.008	0.9304
	CGGGGA	0.109, 0.146	3.268	0.0706
	CAGGCG	0.029, 0.025	0.147	0.7015

**Table 6:** Association of genotype and demographic characteristics in UCP2.

Genotype	Age (year)	BMI (kg/m^2^)	Waist-to-hip ratio	Systolic blood pressure (mmHg)	Diastolic blood pressure (mmHg)
*rs660339*					
GG	52.87 ± 1.03	25.62 ± 0.29	0.94 ± 0.00	129.51 ± 1.61	75.28 ± 0.78
AA	54.22 ± 1.00	25.94 ± 0.35	0.95 ± 0.02	128.74 ± 1.78	76.42 ± 0.96
GA	54.00 ± 0.66	26.07 ± 0.23	0.94 ± 0.00	126.78 ± 1.03	76.62 ± 0.59
*P* value	0.557	0.51	0.492	0.3	0.405
*rs659366*					
CC	53.26 ± 1.03	25.55 ± 0.28	0.94 ± 0.00	129.69 ± 1.65	75.39 ± 0.79
TT	54.48 ± 1.00	25.91 ± 0.34	0.95 ± 0.02	128.60 ± 1.76	76.15 ± 0.93
TC	53.68 ± 0.67	26.11 ± 0.23	0.94 ± 0.00	126.75 ± 1.02	76.67 ± 0.59
*P* value	0.683	0.353	0.503	0.269	0.452
*rs649446*					
CC	52.47 ± 0.78	25.63 ± 0.23	0.94 ± 0.00	128.65 ± 1.18	75.85 ± 0.64
TT	56.44 ± 1.51	26.71 ± 0.48	0.97 ± 0.04	128.98 ± 2.59	74.18 ± 1.41
TC	54.29 ± 0.68	26.02 ± 0.25	0.94 ± 0.00	126.87 ± 1.15	76.91 ± 0.61
*P* value	0.058	0.132	0.055	0.5	0.138
*rs7109266*					
GG	52.61 ± 0.78	25.64 ± 0.23	0.93 ± 0.00	128.65 ± 1.17	75.78 ± 0.64
AA	56.28 ± 1.57	26.65 ± 0.50	0.98 ± 0.04	129.26 ± 2.70	74.69 ± 1.44
GA	54.24 ± 0.68	26.03 ± 0.25	0.94 ± 0.00	126.82 ± 1.14	76.84 ± 0.61
*P* value	0.068	0.177	0.017^∗^	0.456	0.254
*rs591758*					
GG	53.24 ± 1.03	25.59 ± 0.28	0.94 ± 0.00	129.77 ± 1.63	75.56 ± 0.78
CC	54.71 ± 0.99	25.95 ± 0.34	0.95 ± 0.02	128.62 ± 1.76	76.05 ± 0.90
CG	53.57 ± 0.67	26.08 ± 0.24	0.94 ± 0.00	126.64 ± 1.03	76.64 ± 0.60
*P* value	0.539	0.45	0.43	0.224	0.558
*rs586773*					
AA	53.24 ± 1.03	25.59 ± 0.28	0.94 ± 0.00	129.77 ± 1.63	75.56 ± 0.78
TT	54.71 ± 0.99	25.95 ± 0.34	0.95 ± 0.02	128.62 ± 1.76	76.05 ± 0.90
AT	53.64 ± 0.68	26.07 ± 0.24	0.94 ± 0.00	126.67 ± 1.03	76.66 ± 0.61
*P* value	0.553	0.459	0.436	0.235	0.548
*rs34408426*					
AA	53.24 ± 1.03	25.59 ± 0.28	0.94 ± 0.00	129.77 ± 1.63	75.56 ± 0.78
GG	54.71 ± 0.99	25.95 ± 0.34	0.95 ± 0.02	128.62 ± 1.76	76.05 ± 0.90
AG	53.64 ± 0.67	26.06 ± 0.24	0.94 ± 0.00	126.77 ± 1.03	76.74 ± 0.60
*P* value	0.552	0.478	0.43	0.26	0.495
*rs3019463*					
CC	53.24 ± 1.04	25.60 ± 0.28	0.94 ± 0.00	129.84 ± 1.64	75.53 ± 0.79
TT	54.71 ± 1.01	25.98 ± 0.34	0.95 ± 0.02	128.83 ± 1.79	76.04 ± 0.92
TC	53.58 ± 0.68	26.03 ± 0.24	0.94 ± 0.00	126.29 ± 1.03	76.38 ± 0.61
*P* value	0.551	0.519	0.429	0.138	0.709

^∗^ *P* < 0.05.

**Table 7:** Association of genotype and demographic characteristics in PPAR*γ*.

Genotype	Age (year)	BMI (kg/m^2^)	Waist-to-hip ratio	Systolic blood pressure (mmHg)	Diastolic blood pressure (mmHg)
*rs2920503*					
CC	53.99 ± 0.71	26.09 ± 0.22	0.94 ± 0.01	128.06 ± 1.10	76.26 ± 0.57
CT	54.25 ± 0.74	25.69 ± 0.27	0.94 ± 0.00	128.02 ± 1.29	75.99 ± 0.71
TT	50.03 ± 1.54	25.78 ± 0.47	0.94 ± 0.00	126.33 ± 2.44	75.91 ± 1.45
*P* value	0.430	0.502	0.583	0.806	0.946
*rs73813168*					
AA	53.79 ± 0.57	25.92 ± 0.19	0.94 ± 0.01	128.15 ± 0.87	76.04 ± 0.48
GA	53.41 ± 1.00	25.89 ± 0.32	0.94 ± 0.01	127.27 ± 1.82	77.02 ± 0.93
GG	52.25 ± 6.13	28.91 ± 1.87	0.94 ± 0.01	124.75 ± 15.30	69.00 ± 4.65
*P* value	0.92	0.29	0.995	0.845	0.215
*rs79310821*					
GA	54.33 ± 0.67	25.73 ± 0.23	0.94 ± 0.00	127.97 ± 1.06	76.23 ± 0.61
GG	52.11 ± 0.77	26.19 ± 0.25	0.94 ± 0.00	127.57 ± 1.30	76.31 ± 0.69
AA	53.66 ± 1.55	25.77 ± 0.52	0.93 ± 0.01	128.56 ± 2.43	75.95 ± 1.11
*P* value	0.051	0.378	0.735	0.924	0.969
*rs73021485*					
GT	55.29 ± 0.67	25.73 ± 0.23	0.95 ± 0.01	128.21 ± 1.06	76.30 ± 0.61
GG	53.87 ± 0.78	26.17 ± 0.25	0.94 ± 0.00	127.35 ± 1.30	76.17 ± 0.69
TT	54.29 ± 1.53	25.81 ± 0.50	0.93 ± 0.01	128.13 ± 2.38	75.75 ± 1.08
*P* value	0.058	0.425	0.577	0.867	0.923
*rs2920502*					
GC	54.15 ± 0.71	25.93 ± 0.22	0.95 ± 0.01	128.24 ± 1.19	76.62 ± 0.64
CC	53.97 ± 0.72	25.77 ± 0.24	0.93 ± 0.00	127.89 ± 1.12	75.98 ± 0.61
GG	50.36 ± 1.85	26.72 ± 0.61	0.94 ± 0.00	126.33 ± 2.78	75.10 ± 1.43
*P* value	0.098	0.274	0.352	0.803	0.561
*rs17029007*					
GG	53.76 ± 0.57	25.92 ± 0.19	0.94 ± 0.01	128.17 ± 0.86	76.07 ± 0.47
GA	53.74 ± 1.03	25.87 ± 0.34	0.94 ± 0.01	126.59 ± 1.86	77.01 ± 0.96
AA	50.75 ± 4.20	27.74 ± 1.04	0.94 ± 0.01	128.88 ± 10.20	68.75 ± 2.56
*P* value	0.768	0.399	0.992	0.698	0.069
*rs3856806*					
CC	52.84 ± 0.62	25.84 ± 0.20	0.94 ± 0.00	128.17 ± 1.01	76.17 ± 0.53
TC	54.77 ± 0.84	26.03 ± 0.30	0.95 ± 0.01	127.02 ± 1.26	76.35 ± 0.73
TT	55.45 ± 2.47	26.33 ± 0.71	0.93 ± 0.01	130.75 ± 5.31	75.50 ± 2.73
*P* value	0.052	0.765	0.613	0.616	0.932

**Table 8:** Association of genotype with insulin secretion function and blood glucose in UCP2.

Genotype	HbA1c%	FPG (mmol/l)	Glu30′ (mmol/l)	Glu60′ (mmol/l)	Glu120′ (mmol/l)	Ins0′ (*μ*IU/ml)	Ins30′ (*μ*IU/ml)	Ins60′ (*μ*IU/ml)	Ins120′ (*μ*IU/ml)	HOMA-IR	HOMA-*β*	DI_30_	DI_120_
*rs660339*													
GG	6.06 ± 0.11	6.89 ± 0.21	11.37 ± 0.31	11.26 ± 0.42	9.66 ± 0.46	11.79 ± 0.77	78.32 ± 5.24	78.70 ± 4.76	51.97 ± 3.60	3.43 ± 0.23	88.43 ± 5.73	414.74 ± 23.47	536.01 ± 24.80
AA	5.99 ± 0.11	6.80 ± 0.23	11.23 ± 0.32	11.38 ± 0.44	9.55 ± 0.48	12.48 ± 1.16	69.12 ± 4.91	74.85 ± 4.53	57.01 ± 3.97	3.92 ± 0.43	90.93 ± 6.62	383.99 ± 27.42	514.93 ± 28.03
GA	6.01 ± 0.07	6.68 ± 0.13	11.01 ± 0.21	10.92 ± 0.29	8.83 ± 0.28	12.29 ± 0.61	71.72 ± 3.28	78.81 ± 3.47	58.45 ± 3.07	3.97 ± 0.33	89.23 ± 3.38	420.51 ± 17.56	587.96 ± 34.53
*P* value	0.894	0.677	0.592	0.633	0.187	0.849	0.379	0.803	0.412	0.524	0.95	0.518	0.294
*rs659366*													
CC	6.05 ± 0.11	6.88 ± 0.22	11.37 ± 0.32	11.28 ± 0.43	9.68 ± 0.47	11.80 ± 0.79	77.38 ± 5.27	78.68 ± 4.82	52.52 ± 3.68	3.43 ± 0.24	88.80 ± 5.86	410.35 ± 23.43	535.63 ± 25.18
TT	5.93 ± 0.11	6.74 ± 0.22	11.09 ± 0.31	11.07 ± 0.43	9.31 ± 0.46	12.56 ± 1.12	71.34 ± 5.05	75.55 ± 4.77	58.35 ± 4.31	3.88 ± 0.42	92.55 ± 6.54	395.63 ± 27.34	526.68 ± 27.42
TC	6.04 ± 0.07	6.71 ± 0.14	11.07 ± 0.21	11.04 ± 0.29	8.92 ± 0.28	12.24 ± 0.60	71.39 ± 3.25	78.60 ± 3.41	57.57 ± 2.98	3.99 ± 0.33	88.32 ± 3.34	418.18 ± 17.59	583.49 ± 34.57
*P* value	0.677	0.796	0.706	0.889	0.327	0.84	0.56	0.871	0.526	0.518	0.821	0.777	0.43
*rs649446*													
CC	6.06 ± 0.09	6.84 ± 0.16	11.24 ± 0.24	11.20 ± 0.33	9.46 ± 0.34	12.05 ± 0.55	75.14 ± 3.99	79.96 ± 3.95	56.72 ± 3.32	3.61 ± 0.18	90.91 ± 4.38	404.52 ± 18.16	535.54 ± 20.23
TT	6.06 ± 0.16	6.90 ± 0.32	11.50 ± 0.47	11.65 ± 0.65	10.06 ± 0.73	16.71 ± 2.96	74.87 ± 7.74	83.49 ± 7.86	67.33 ± 7.15	5.78 ± 1.53	108.66 ± 12.90	335.15 ± 26.95	466.10 ± 31.42
TC	5.98 ± 0.07	6.67 ± 0.15	11.01 ± 0.21	10.93 ± 0.29	8.81 ± 0.30	11.38 ± 0.50	70.59 ± 3.32	75.06 ± 3.24	54.06 ± 2.76	3.59 ± 0.23	83.77 ± 3.10	433.37 ± 19.96	599.24 ± 37.91
*P* value	0.765	0.676	0.581	0.567	0.156	0.003^∗^	0.655	0.469	0.176	0.006^∗^	0.026^∗^	0.066	0.096
*rs7109266*													
GG	6.07 ± 0.09	6.86 ± 0.16	11.24 ± 0.24	11.24 ± 0.33	9.48 ± 0.34	12.03 ± 0.55	74.80 ± 3.96	79.68 ± 3.92	56.77 ± 3.29	3.62 ± 0.18	90.40 ± 4.35	402.30 ± 18.07	533.33 ± 20.14
AA	6.07 ± 0.17	6.96 ± 0.33	11.58 ± 0.50	11.79 ± 0.67	10.16 ± 0.77	17.11 ± 3.12	76.19 ± 8.10	84.59 ± 8.20	68.44 ± 7.51	5.98 ± 1.61	109.45 ± 13.52	334.04 ± 28.21	461.60 ± 33.00
GA	5.97 ± 0.07	6.64 ± 0.15	10.99 ± 0.21	10.87 ± 0.29	8.78 ± 0.30	11.37 ± 0.49	70.64 ± 3.31	75.15 ± 3.23	53.93 ± 2.75	3.57 ± 0.23	84.30 ± 3.09	434.69 ± 19.84	601.13 ± 37.71
*P* value	0.601	0.502	0.49	0.401	0.107	0.001^∗^	0.656	0.449	0.137	0.003^∗^	0.031^∗^	0.06	0.078
*rs591758*													
GG	6.05 ± 0.11	6.87 ± 0.21	11.33 ± 0.31	11.24 ± 0.42	9.66 ± 0.46	11.91 ± 0.79	77.66 ± 5.22	78.40 ± 4.75	52.65 ± 3.63	3.46 ± 0.23	89.39 ± 5.7	410.86 ± 23.20	534.33 ± 24.89
CC	5.94 ± 0.11	6.78 ± 0.23	11.11 ± 0.30	11.11 ± 0.41	9.32 ± 0.45	13.43 ± 1.36	73.30 ± 5.06	78.32 ± 4.83	59.57 ± 4.33	4.45 ± 0.70	94.35 ± 6.42	392.34 ± 26.39	525.85 ± 26.71
CG	6.04 ± 0.07	6.70 ± 0.14	11.07 ± 0.21	11.04 ± 0.29	8.91 ± 0.29	11.79 ± 0.48	70.32 ± 3.25	77.56 ± 3.43	56.98 ± 3.00	3.71 ± 0.21	87.12 ± 3.36	419.88 ± 17.95	585.93 ± 35.37
*P* value	0.73	0.775	0.77	0.92	0.343	0.322	0.457	0.987	0.485	0.209	0.57	0.682	0.381
*rs586773*													
AA	6.05 ± 0.11	6.87 ± 0.21	11.33 ± 0.31	11.24 ± 0.42	9.66 ± 0.46	11.91 ± 0.79	77.66 ± 5.22	78.40 ± 4.75	52.65 ± 3.63	3.46 ± 0.23	89.39 ± 5.79	410.86 ± 23.20	534.33 ± 24.89
TT	5.94 ± 0.11	6.78 ± 0.23	11.11 ± 0.30	11.11 ± 0.41	9.32 ± 0.45	13.43 ± 1.36	73.30 ± 5.06	78.32 ± 4.83	59.57 ± 4.33	4.45 ± 0.70	94.35 ± 6.42	392.34 ± 26.39	525.85 ± 26.71
AT	6.04 ± 0.07	6.71 ± 0.14	11.10 ± 0.21	11.09 ± 0.30	8.93 ± 0.29	11.80 ± 0.49	70.25 ± 3.29	77.60 ± 3.46	57.04 ± 3.03	3.73 ± 0.22	86.95 ± 3.39	418.26 ± 18.06	583.80 ± 35.73
*P* value	0.711	0.804	0.798	0.952	0.365	0.33	0.453	0.988	0.484	0.214	0.557	0.713	0.413
*rs34408426*													
AA	6.05 ± 0.11	6.87 ± 0.21	11.33 ± 0.31	11.24 ± 0.42	9.66 ± 0.46	11.91 ± 0.79	77.66 ± 5.22	78.40 ± 4.75	52.65 ± 3.63	3.46 ± 0.23	89.39 ± 5.79	410.86 ± 23.20	534.33 ± 24.89
GG	5.94 ± 0.11	6.78 ± 0.23	11.11 ± 0.30	11.11 ± 0.41	9.32 ± 0.45	13.43 ± 1.36	73.30 ± 5.06	78.32 ± 4.83	59.57 ± 4.33	4.45 ± 0.70	94.35 ± 6.42	392.34 ± 26.39	525.85 ± 26.71
AG	6.03 ± 0.07	6.67 ± 0.13	11.02 ± 0.21	11.00 ± 0.29	8.90 ± 0.29	11.81 ± 0.49	70.71 ± 3.28	77.99 ± 3.46	57.37 ± 3.02	3.71 ± 0.21	87.48 ± 3.38	421.79 ± 18.08	588.64 ± 35.70
*P* value	0.747	0.698	0.694	0.882	0.331	0.332	0.499	0.997	0.471	0.209	0.602	0.646	0.35
*rs3019463*													
CC	6.03 ± 0.11	6.85 ± 0.21	11.30 ± 0.31	11.19 ± 0.42	9.60 ± 0.46	11.91 ± 0.79	77.96 ± 5.25	78.59 ± 4.78	52.63 ± 3.66	3.45 ± 0.24	89.77 ± 5.82	413.23 ± 23.25	537.30 ± 24.89
TT	5.96 ± 0.11	6.82 ± 0.23	11.15 ± 0.31	11.23 ± 0.42	9.40 ± 0.46	13.54 ± 1.39	72.78 ± 5.11	79.20 ± 4.90	60.35 ± 4.41	4.51 ± 0.72	94.20 ± 6.53	381.46 ± 25.95	518.95 ± 26.89
TC	6.02 ± 0.07	6.67 ± 0.14	11.08 ± 0.22	11.00 ± 0.30	8.91 ± 0.29	11.75 ± 0.50	70.74 ± 3.30	78.34 ± 3.50	57.65 ± 3.08	3.70 ± 0.22	87.13 ± 3.38	425.19 ± 18.44	593.35 ± 36.28
*P* value	0.864	0.74	0.847	0.883	0.369	0.278	0.473	0.99	0.41	0.172	0.59	0.39	0.275

^∗^ *P* < 0.05. DI_30_ (early-phase disposition index of insulin secretion) = [InsAUC_30_/GluAUC_30_] × ISI_M_, DI_120_ (total-phase disposition of insulin secretion) = [InsAUC_120_/GluAUC_120_] × ISI_M_.

**Table 9:** Association of genotype with lipid profiles in UCP2.

Genotype	TC (mmol/l)	TG (mmol/l)	HDL-C (mmol/l)	LDL-C (mmol/l)	TG/HDL-C
*rs660339*					
GG	5.44 ± 0.09	1.81 ± 0.16	1.35 ± 0.04	2.82 ± 0.06	1.45 ± 0.12
AA	5.49 ± 0.10	1.72 ± 0.10	1.30 ± 0.03	2.85 ± 0.07	1.45 ± 0.10
GA	5.39 ± 0.06	2.16 ± 0.41	1.29 ± 0.02	2.83 ± 0.04	1.79 ± 0.32
*P* value	0.702	0.678	0.359	0.93	0.627
*rs659366*					
CC	5.44 ± 0.09	1.73 ± 0.14	1.34 ± 0.05	2.83 ± 0.06	1.41 ± 0.11
TT	5.49 ± 0.10	1.69 ± 0.10	1.30 ± 0.03	2.86 ± 0.07	1.43 ± 0.09
TC	5.39 ± 0.06	2.22 ± 0.42	1.29 ± 0.02	2.82 ± 0.04	1.82 ± 0.32
*P* value	0.672	0.536	0.405	0.919	0.519
*rs649446*					
CC	5.35 ± 0.07	2.34 ± 0.53	1.34 ± 0.03	2.77 ± 0.05	1.85 ± 0.40
TT	5.61 ± 0.16	1.66 ± 0.12	1.29 ± 0.04	2.95 ± 0.12	1.40 ± 0.12
TC	5.44 ± 0.06	1.72 ± 0.07	1.28 ± 0.02	2.86 ± 0.05	1.48 ± 0.07
*P* value	0.254	0.39	0.257	0.157	0.544
*rs7109266*					
GG	5.35 ± 0.07	2.33 ± 0.52	1.34 ± 0.03	2.77 ± 0.05	1.84 ± 0.40
AA	5.56 ± 0.17	1.63 ± 0.12	1.28 ± 0.04	2.93 ± 0.12	1.39 ± 0.13
GA	5.45 ± 0.06	1.73 ± 0.07	1.28 ± 0.02	2.87 ± 0.04	1.48 ± 0.07
*P* value	0.351	0.404	0.249	0.206	0.563
*rs591758*					
GG	5.44 ± 0.09	1.72 ± 0.13	1.34 ± 0.04	2.84 ± 0.06	1.41 ± 0.11
CC	5.49 ± 0.10	1.69 ± 0.10	1.30 ± 0.03	2.86 ± 0.07	1.43 ± 0.09
CG	5.38 ± 0.06	2.23 ± 0.43	1.29 ± 0.02	2.82 ± 0.04	1.83 ± 0.33
*P* value	0.633	0.509	0.497	0.846	0.501
*rs586773*					
AA	5.44 ± 0.09	1.72 ± 0.13	1.34 ± 0.04	2.84 ± 0.06	1.41 ± 0.11
TT	5.49 ± 0.10	1.69 ± 0.10	1.30 ± 0.03	2.86 ± 0.07	1.43 ± 0.09
AT	5.38 ± 0.06	2.23 ± 0.43	1.30 ± 0.02	2.82 ± 0.04	1.82 ± 0.33
*P* value	0.635	0.515	0.515	0.855	0.508
*rs34408426*					
AA	5.44 ± 0.09	1.72 ± 0.13	1.34 ± 0.04	2.84 ± 0.06	1.41 ± 0.11
GG	5.49 ± 0.10	1.69 ± 0.10	1.30 ± 0.03	2.86 ± 0.07	1.43 ± 0.09
AG	5.37 ± 0.06	2.20 ± 0.43	1.29 ± 0.02	2.81 ± 0.04	1.82 ± 0.33
*P* value	0.536	0.554	0.449	0.829	0.521
*rs3019463*					
CC	5.43 ± 0.09	1.72 ± 0.14	1.34 ± 0.05	2.83 ± 0.06	1.41 ± 0.11
TT	5.51 ± 0.10	1.69 ± 0.10	1.30 ± 0.03	2.87 ± 0.07	1.43 ± 0.09
TC	5.38 ± 0.07	2.23 ± 0.44	1.30 ± 0.02	2.81 ± 0.04	1.82 ± 0.34
*P* value	0.538	0.522	0.561	0.744	0.527

**Table 10:** Association of genotype with insulin secretion function and blood glucose in PPAR*γ*.

Genotype	HbA1c%	FPG (mmol/l)	Glu30′ (mmol/l)	Glu60′ (mmol/l)	Glu120′ (mmol/l)	Ins0′ (*μ*IU/ml)	Ins30′ (*μ*IU/ml)	Ins60′ (*μ*IU/ml)	Ins120′ (*μ*IU/ml)	HOMA-IR	HOMA-*β*	DI_30_	DI_120_
*rs2920503*													
CC	6.06 ± 0.08	6.84 ± 0.16	11.34 ± 0.23	11.16 ± 0.32	9.17 ± 0.33	11.59 ± 0.57	73.21 ± 3.49	74.50 ± 3.44	50.88 ± 2.74	3.69 ± 0.33	85.26 ± 3.25	423.09 ± 18.98	577.78 ± 37.56
CT	6.10 ± 0.08	6.87 ± 0.16	11.32 ± 0.25	11.55 ± 0.33	9.69 ± 0.35	13.10 ± 0.86	72.05 ± 3.91	82.18 ± 3.95	53.29 ± 6.52	4.15 ± 0.32	92.27 ± 5.27	387.24 ± 19.31	527.33 ± 20.78
TT	5.59 ± 0.11	5.98 ± 0.12	9.82 ± 0.29	9.33 ± 0.44	7.72 ± 0.35	12.05 ± 0.92	78.26 ± 8.06	78.79 ± 6.95	62.76 ± 3.49	3.31 ± 0.29	101.01 ± 6.49	460.64 ± 35.70	592.80 ± 31.89
*P* value	0.019^∗^	0.039^∗^	0.012^∗^	0.01^∗^	0.034^∗^	0.295	0.77	0.332	0.024^∗^	0.405	0.186	0.178	0.434
*rs73813168*													
AA	6.02 ± 0.06	6.77 ± 0.12	11.16 ± 0.18	11.14 ± 0.24	9.32 ± 0.25	11.90 ± 0.41	71.68 ± 2.77	78.64 ± 2.76	57.65 ± 2.39	3.67 ± 0.17	87.72 ± 2.87	402.67 ± 13.84	556.93 ± 24.92
GA	6.04 ± 0.11	6.78 ± 0.23	11.21 ± 0.32	11.13 ± 0.46	8.87 ± 0.46	12.14 ± 1.04	75.88 ± 5.27	76.82 ± 5.22	52.35 ± 4.13	3.99 ± 0.63	89.11 ± 5.46	439.29 ± 29.79	570.00 ± 31.05
GG	5.85 ± 0.12	5.99 ± 0.19	11.09 ± 0.80	10.54 ± 1.91	6.21 ± 1.12	12.59 ± 3.34	99.09 ± 51.54	73.55 ± 20.46	43.04 ± 15.77	3.35 ± 0.86	102.59 ± 30.85	414.77 ± 154.51	513.87 ± 66.05
*P* value	0.947	0.814	0.99	0.97	0.336	0.96	0.506	0.939	0.479	0.777	0.864	0.477	0.946
*rs79310821*													
GA	6.16 ± 0.08	7.08 ± 0.18	11.59 ± 0.24	11.76 ± 0.33	9.92 ± 0.35	12.81 ± 0.87	68.22 ± 3.24	76.64 ± 3.47	56.29 ± 3.03	3.92 ± 0.36	83.64 ± 3.79	412.36 ± 20.56	522.64 ± 21.28
GG	5.91 ± 0.08	6.52 ± 0.13	10.80 ± 0.22	10.68 ± 0.31	8.71 ± 0.31	12.14 ± 0.48	76.31 ± 3.96	78.00 ± 3.63	56.77 ± 3.09	3.80 ± 0.28	91.79 ± 4.26	403.67 ± 16.44	586.01 ± 36.88
AA	5.89 ± 0.11	6.39 ± 0.17	10.67 ± 0.37	10.13 ± 0.51	8.30 ± 0.52	10.14 ± 0.62	78.61 ± 8.39	80.96 ± 8.19	56.84 ± 6.70	3.50 ± 0.33	105.43 ± 8.54	442.89 ± 46.25	583.89 ± 43.95
*P* value	0.055	0.015^∗^	0.029^∗^	0.012^∗^	0.009^∗^	0.192	0.21	0.856	0.993	0.855	0.069	0.695	0.301
*rs73021485*													
GT	6.14 ± 0.08	7.06 ± 0.18	11.59 ± 0.24	11.74 ± 0.32	9.86 ± 0.34	12.32 ± 0.82	68.11 ± 3.22	76.83 ± 3.47	56.48 ± 3.03	4.14 ± 0.40	82.09 ± 3.90	373.32 ± 17.21	509.27 ± 19.15
GG	5.91 ± 0.08	6.51 ± 0.13	10.80 ± 0.22	10.67 ± 0.31	8.67 ± 0.31	12.17 ± 0.48	76.73 ± 3.97	78.64 ± 3.66	56.42 ± 3.09	3.56 ± 0.17	96.67 ± 4.06	438.29 ± 20.28	564.71 ± 19.91
TT	5.97 ± 0.12	6.51 ± 0.19	10.74 ± 0.39	10.30 ± 0.55	8.59 ± 0.57	11.87 ± 1.25	78.18 ± 8.14	80.13 ± 7.86	56.92 ± 6.44	3.52 ± 0.48	91.36 ± 9.05	460.55 ± 37.27	724.86 ± 133.17I
*P* value	0.112	0.028^∗^	0.031^∗^	0.019^∗^	0.021^∗^	0.949	0.186	0.89	0.997	0.363	0.041^∗^	0.018^∗^	0.004^∗^
*rs2920502*													
GC	6.20 ± 0.10	7.11 ± 0.19	11.58 ± 0.26	11.67 ± 0.35	8.98 ± 0.28	11.75 ± 0.64	71.26 ± 3.88	73.99 ± 3.59	54.91 ± 5.56	3.56 ± 0.17	84.54 ± 4.31	436.29 ± 20.24	561.96 ± 19.83
CC	6.02 ± 0.06	7.15 ± 0.11	11.85 ± 0.19	11.76 ± 0.27	9.76 ± 0.38	12.55 ± 0.73	73.30 ± 3.47	70.05 ± 3.57	50.38 ± 2.98	4.33 ± 0.42	96.17 ± 4.05	373.56 ± 17.36	507.65 ± 19.37
GG	5.74 ± 0.11	6.25 ± 0.24	10.72 ± 0.46	10.43 ± 0.64	7.82 ± 0.52	12.68 ± 1.00	80.64 ± 7.31	85.24 ± 7.56	62.44 ± 3.16	2.81 ± 0.19	83.97 ± 6.40	470.32 ± 37.73	743.78 ± 136.80
*P* value	0.009^∗^	0.01^∗^	0.049^∗^	0.068	0.032^∗^	0.663	0.579	0.015^∗^	0.019^∗^	0.041^∗^	0.104	0.015^∗^	0.002^∗^
*rs17029007*													
GG	6.01 ± 0.06	6.77 ± 0.12	11.14 ± 0.18	11.12 ± 0.24	9.30 ± 0.25	12.18 ± 0.50	71.74 ± 2.75	78.71 ± 2.74	57.73 ± 2.40	3.76 ± 0.19	89.10 ± 3.15	402.92 ± 13.76	557.25 ± 24.78
GA	6.00 ± 0.11	6.76 ± 0.24	11.23 ± 0.33	11.16 ± 0.48	8.98 ± 0.49	12.35 ± 1.09	75.56 ± 5.32	76.13 ± 5.39	52.52 ± 4.26	4.05 ± 0.65	90.98 ± 5.74	437.31 ± 30.27	563.96 ± 31.32
AA	6.26 ± 0.50	6.01 ± 0.16	10.68 ± 0.62	9.78 ± 1.21	6.35 ± 0.79	10.79 ± 1.81	111.10 ± 30.97	79.93 ± 13.81	49.28 ± 10.87	2.87 ± 0.47	89.40 ± 17.08	514.60 ± 108.50	642.35 ± 83.52
*P* value	0.837	0.684	0.903	0.741	0.231	0.92	0.138	0.904	0.532	0.725	0.96	0.319	0.873
*rs3856806*													
CC	5.96 ± 0.06	6.57 ± 0.11	10.92 ± 0.18	10.78 ± 0.25	8.93 ± 0.26	11.87 ± 0.41	75.61 ± 3.14	77.37 ± 3.07	56.12 ± 2.58	3.51 ± 0.14	91.40 ± 3.05	432.97 ± 16.46	582.14 ± 29.37
TC	6.12 ± 0.10	7.11 ± 0.22	11.58 ± 0.29	11.68 ± 0.39	9.69 ± 0.40	12.75 ± 1.08	69.35 ± 4.21	78.76 ± 4.16	56.36 ± 3.54	4.33 ± 0.53	86.40 ± 5.67	377.68 ± 20.08	518.92 ± 22.77
TT	6.08 ± 0.21	7.88 ± 0.47	11.33 ± 0.82	11.60 ± 1.24	9.27 ± 1.42	12.59 ± 2.01	62.15 ± 8.81	77.54 ± 11.07	58.24 ± 12.15	4.36 ± 1.22	82.71 ± 11.35	358.63 ± 57.56	506.31 ± 62.29
*P* value	0.326	0.047^∗^	0.127	0.121	0.257	0.65	0.338	0.964	0.981	0.155	0.619	0.085	0.302

^∗^ *P* < 0.05. DI_30_ (early-phase disposition index of insulin secretion) = [InsAUC_30_/GluAUC_30_] × ISI_M_, DI_120_ (total-phase disposition of insulin secretion) = [InsAUC_120_/GluAUC_120_] × ISI_M_.

**Table 11:** Association of genotype with lipid profiles in PPAR*γ*.

Genotype	TC (mmol/l)	TG (mmol/l)	HDL-C (mmol/l)	LDL-C (mmol/l)	TG/HDL-C
*rs2920503*					
CC	5.51 ± 0.07	2.25 ± 0.46	1.32 ± 0.03	2.87 ± 0.05	1.81 ± 0.35
CT	5.36 ± 0.08	1.72 ± 0.10	1.28 ± 0.02	2.83 ± 0.05	1.44 ± 0.08
TT	5.26 ± 0.11	1.78 ± 0.16	1.33 ± 0.08	2.66 ± 0.08	1.56 ± 0.17
*P* value	0.154	0.53	0.457	0.146	0.594
*rs73813168*					
AA	5.37 ± 0.05	1.78 ± 0.08	1.29 ± 0.02	2.80 ± 0.03	1.49 ± 0.06
GA	5.44 ± 0.11	2.67 ± 0.94	1.37 ± 0.05	2.97 ± 0.08	2.11 ± 0.72
GG	5.26 ± 0.59	1.36 ± 0.23	1.27 ± 0.13	2.73 ± 0.44	1.06 ± 0.12
*P* value	0.054	0.245	0.164	0.057	0.313
*rs79310821*					
GA	5.49 ± 0.06	1.70 ± 0.08	1.30 ± 0.02	2.88 ± 0.04	1.41 ± 0.07
GG	5.42 ± 0.08	1.81 ± 0.11	1.33 ± 0.03	2.83 ± 0.05	1.49 ± 0.09
AA	5.20 ± 0.12	1.88 ± 0.24	1.25 ± 0.03	2.67 ± 0.09	1.61 ± 0.18
*P* value	0.147	0.629	0.283	0.109	0.484
*rs73021485*					
GT	5.49 ± 0.06	1.70 ± 0.08	1.29 ± 0.02	2.88 ± 0.04	1.41 ± 0.07
GG	5.41 ± 0.08	2.30 ± 0.52	1.33 ± 0.03	2.83 ± 0.05	1.87 ± 0.40
TT	5.19 ± 0.12	1.85 ± 0.23	1.26 ± 0.03	2.67 ± 0.09	1.58 ± 0.18
*P* value	0.144	0.452	0.313	0.109	0.464
*rs2920502*					
GC	5.48 ± 0.07	2.39 ± 0.08	1.31 ± 0.02	2.90 ± 0.05	1.82 ± 0.08
CC	5.72 ± 0.20	4.35 ± 2.43	1.28 ± 0.02	2.98 ± 0.15	3.40 ± 0.07
GG	5.32 ± 0.06	1.73 ± 0.11	1.42 ± 0.12	2.75 ± 0.04	1.22 ± 0.26
*P* value	0.034^∗^	0.004^∗∗^	0.07	0.031^∗^	0.006^∗∗^
*rs17029007*					
GG	5.37 ± 0.05	1.78 ± 0.07	1.29 ± 0.02	2.80 ± 0.03	1.49 ± 0.06
GA	5.58 ± 0.10	2.70 ± 0.98	1.38 ± 0.05	2.91 ± 0.07	2.13 ± 0.75
AA	5.55 ± 0.45	1.46 ± 0.23	1.29 ± 0.09	2.94 ± 0.31	1.15 ± 0.17
*P* value	0.165	0.233	0.071	0.334	0.301
*rs3856806*					
CC	5.42 ± 0.06	2.04 ± 0.35	1.30 ± 0.02	2.84 ± 0.04	1.71 ± 0.27
TC	5.44 ± 0.08	1.91 ± 0.16	1.32 ± 0.02	2.81 ± 0.05	1.52 ± 0.11
TT	5.39 ± 0.27	1.48 ± 0.17	1.25 ± 0.06	2.88 ± 0.16	1.27 ± 0.16
*P* value	0.971	0.882	0.741	0.846	0.811

^∗^ *P* < 0.05 and ^∗∗^ *P* < 0.01.

**Table 12:** Association of genotype with inflammation in UCP2.

Genotype	TNF-*α* (fmol/ml)	IL-6 (pg/ml)
*rs660339*		
GG	23.44 ± 0.86	1.62 ± 0.08
AA	22.25 ± 0.95	1.42 ± 0.10
GA	22.40 ± 0.57	1.70 ± 0.05
*P* value	0.527	0.034^∗^
*rs659366*		
CC	23.31 ± 0.87	1.64 ± 0.08
TT	22.10 ± 0.94	1.46 ± 0.10
TC	22.55 ± 0.57	1.67 ± 0.05
*P* value	0.598	0.111
*rs649446*		
CC	23.28 ± 0.66	1.68 ± 0.06
TT	20.97 ± 1.48	1.95 ± 0.16
TC	22.37 ± 0.59	1.65 ± 0.06
*P* value	0.257	0.001^∗∗^
*rs7109266*		
GG	23.22 ± 0.66	1.68 ± 0.06
AA	20.78 ± 1.50	1.52 ± 0.17
GA	22.45 ± 0.60	1.65 ± 0.06
*P* value	0.257	0.063
*rs591758*		
GG	23.37 ± 0.86	1.64 ± 0.08
CC	21.81 ± 0.89	1.46 ± 0.10
CG	22.65 ± 0.59	1.68 ± 0.06
*P* value	0.439	0.111
*rs586773*		
AA	23.37 ± 0.86	1.64 ± 0.08
TT	21.81 ± 0.89	1.46 ± 0.10
AT	22.61 ± 0.59	1.67 ± 0.06
*P* value	0.437	0.12
*rs34408426*		
AA	23.37 ± 0.86	1.64 ± 0.08
GG	21.81 ± 0.89	1.46 ± 0.10
AG	22.56 ± 0.59	1.67 ± 0.06
*P* value	0.434	0.124
*rs3019463*		
CC	23.34 ± 0.86	1.64 ± 0.08
TT	22.15 ± 0.89	1.47 ± 0.10
TC	22.58 ± 0.60	1.66 ± 0.06
*P* value	0.606	0.17

^∗^ *P* < 0.05 and ^∗∗^ *P* < 0.01. TNF-*α*: tumor necrosis factor-*α*; IL-6: interleukine-6. IL-6 has been nature logarithm transformed.

**Table 13:** Association of genotype with inflammation in PPAR*γ*.

Genotype	TNF-*α* (fmol/ml)	IL-6 (pg/ml)
*rs2920503*		
CC	22.60 ± 0.64	57.77 ± 0.96
CT	22.42 ± 0.62	60.82 ± 1.02
TT	23.51 ± 1.51	60.76 ± 2.40
*P* value	0.778	0.081
*rs73813168*		
AA	23.11 ± 0.49	1.63 ± 0.05
GA	21.68 ± 0.88	1.56 ± 0.09
GG	16.44 ± 4.27	1.76 ± 0.27
*P* value	0.164	0.742
*rs79310821*		
GA	23.02 ± 0.63	1.64 ± 0.06
GG	22.34 ± 0.66	1.59 ± 0.07
AA	22.29 ± 1.25	1.64 ± 0.11
*P* value	0.723	0.844
*rs73021485*		
GT	22.99 ± 0.63	1.64 ± 0.06
GG	22.32 ± 0.67	1.59 ± 0.07
TT	22.28 ± 1.22	1.60 ± 0.11
*P* value	0.735	0.81
*rs2920502*		
GC	22.78 ± 0.67	1.57 ± 0.06
CC	22.70 ± 0.61	1.68 ± 0.06
GG	21.41 ± 1.41	1.45 ± 0.14
*P* value	0.665	0.231
*rs17029007*		
GG	23.03 ± 0.49	1.63 ± 0.05
GA	21.70 ± 0.87	1.55 ± 0.10
AA	17.39 ± 3.82	1.80 ± 0.22
*P* value	0.137	0.634
*rs3856806*		
CC	22.62 ± 0.52	1.58 ± 0.05
TC	22.63 ± 0.79	1.69 ± 0.07
TT	21.87 ± 2.18	1.58 ± 0.24
*P* value	0.949	0.419

TNF-*α*: tumor necrosis factor-*α*; IL-6: interleukine-6. IL-6 has been nature logarithm transformed.

## Supplementary Materials

Supplementary Materials Table 1: Hardy-Weinberg equilibrium test of loci in UCP2 and PPAR*γ*.
