# Supplementary material for: Uncoupling Protein 2 and Peroxisome Proliferator-Activated Receptor γ Gene Polymorphisms in Association with Diabetes Susceptibility in Chinese Han Population with Variant Glucose Tolerance
Source: Int J Endocrinol. 2018 Apr 5;2018:4636783. doi: 10.1155/2018/4636783 (PMC5907424; doi:10.1155/2018/4636783)
Supplement: Supplementary Materials — Table 1: Hardy-Weinberg equilibrium test of loci in UCP2 and PPARγ. [file 4636783.f1.pdf]

Supplementary Table 1. Hardy-Weinberg equilibrium test of loci in UCP2 and PPARγ

Gene name	SNP number	Group	Observed heterozygosity	Expectation heterozygosity	*P* value
UCP2	rs660339	Pre-diabetes/Diabetes	0.523	0.498	0.402
		Normal blood glucose	0.520	0.499	0.572
	rs659366	Pre-diabetes/Diabetes	0.520	0.499	0.464
		Normal blood glucose	0.515	0.500	0.776
	rs649446	Pre-diabetes/Diabetes	0.458	0.444	0.638
		Normal blood glucose	0.495	0.451	0.208
	rs586773	Pre-diabetes/Diabetes	0.506	0.499	0.835
		Normal blood glucose	0.5	0.500	1
	rs34408426	Pre-diabetes/Diabetes	0.504	0.499	0.917
		Normal blood glucose	0.503	0.500	1
	rs7109266	Pre-diabetes/Diabetes	0.458	0.440	0.479
		Normal blood glucose	0.5	0.450	0.154
	rs3019463	Pre-diabetes/Diabetes	0.5	0.499	1
		Normal blood glucose	0.505	0.500	1
	rs591758	Pre-diabetes/Diabetes	0.507	0.499	0.834
		Normal blood glucose	0.505	0.500	1
PPARγ	rs2920503	Pre-diabetes/Diabetes	0.407	0.419	0.613
		Normal blood glucose	0.404	0.434	0.329
	rs73813168	Pre-diabetes/Diabetes	0.209	0.196	0.285
		Normal blood glucose	0.276	0.252	0.263
	rs79310821	Pre-diabetes/Diabetes	0.484	0.462	0.638
		Normal blood glucose	0.408	0.436	0.208
	rs73021485	Pre-diabetes/Diabetes	0.482	0.436	0.575
		Normal blood glucose	0.409	0.426	0.415
	rs2920502	Pre-diabetes/Diabetes	0.449	0.414	0.130
		Normal blood glucose	0.416	0.446	0.342
	rs17029007	Pre-diabetes/Diabetes	0.196	0.198	0.790
		Normal blood glucose	0.273	0.257	0.580
	rs3856806	Pre-diabetes/Diabetes	0.359	0.347	0.650
		Normal blood glucose	0.293	0.278	0.610
